# “Now that you are circumcised, you cannot have first sex with your wife”: post circumcision sexual behaviours and beliefs among men in Wakiso district, Uganda

**DOI:** 10.7448/IAS.20.1.21498

**Published:** 2017-06-05

**Authors:** Simon Peter Sebina Kibira, Lynn Muhimbuura Atuyambe, Ingvild Fossgard Sandøy, Fredrick Edward Makumbi, Marguerite Daniel

**Affiliations:** ^a^ Centre for International Health, Department of Global Public Health and Primary Care, University of Bergen, Bergen, Norway; ^b^ Department of Community Health and Behavioural Sciences, School of Public Health, Makerere University, Kampala, Uganda; ^c^ Department of Epidemiology and Biostatistics, School of Public Health, Makerere University, Kampala, Uganda; ^d^ Department of Health Promotion and Development, University of Bergen, Bergen, Norway

**Keywords:** Male circumcision, Sexual risk behaviours, HIV, Uganda

## Abstract

**Introduction**: Safe male circumcision is an important biomedical intervention in the comprehensive HIV prevention programmes implemented in 14 sub-Saharan African countries with high HIV prevalence. To sustain its partial protective benefit, it is important that perceived reduced HIV risk does not lead to behavioural risk compensation among circumcised men and their sexual partners. This study explored beliefs that may influence post circumcision sexual behaviours among circumcised men in a programme setting.

**Methods**: Forty-eight in-depth interviews were conducted with newly circumcised men in Wakiso district, central Uganda. Twenty-five men seeking circumcision services at public health facilities in the district were recruited from May to June 2015 and, interviewed at baseline and after 6 months. Participants’ beliefs and sexual behaviours were compared just after circumcision and at follow up to explore changes. Data were managed using atlas.ti7 and analysed following a thematic network analysis framework.

**Results**: Four themes following safe male circumcision emerged from this study. Beliefs related to: (1) sexual cleansing, (2) healing, (3) post SMC sexual capabilities and (4) continued HIV transmission risk. Most men maintained or adopted safer sexual behaviour; being faithful to their partner after circumcision or using condoms with extramarital partners following the knowledge that there was continued HIV risk post circumcision. The most prevalent risky belief was regarding sexual cleansing post circumcision, and as a result of this belief, some men had one off condom-less sexual intercourse with a casual partner. Some resumed sex before the recommended period due to misunderstanding of what comprised healing.

**Conclusions**: Although most men maintained or adopted safer sexual behaviour, there were instances of risky sexual behaviour resulting from beliefs regarding the first sexual intercourse after circumcision or misunderstandings of what comprised wound healing. If not addressed, these may attenuate the safe male circumcision benefits of risk reduction for HIV.

## Introduction

Male circumcision is an important biomedical intervention that reduces heterosexual HIV infection risk from infected women to men [1–3]. Modelling studies indicate that the direct reduction of HIV transmission to men will also reduce the long-term infection risk to women [4]. Uganda is one of the 14 WHO priority countries [5,6] with epidemic HIV which are implementing the Safe Male Circumcision (SMC) programme. Over two million men have been circumcised under the national SMC programme between 2010 and 2014 [7].

Mixed findings regarding post SMC sexual behaviour have been reported in the clinical trials [1–3] that informed the WHO recommendation for SMC [5]. More sexual partners [2] were reported in the South African trial among the circumcised than the uncircumcised men. In the Kenyan trial, circumcised men exhibited slightly higher sexual risk behaviours than the uncircumcised after a 24-month period of follow up. Fifty-one per cent of the circumcised men reported condom-less sex versus 46% in the control group [3]. However, there were no significant differences in sexual behaviour in the first two years among intervention and control groups in the Ugandan trial [1]. The Ugandan post-trial follow-up studies also reported no evidence of behavioural risk compensation [8,9]. In a programme setting in Kisumu, Kenya, Westercamp and colleagues also observed no evidence of risk compensation [10]. They instead found that both circumcised and uncircumcised men exposed to the SMC programme and information messages, respectively, adopted safer sexual behaviours. Such findings are reassuring, but contexts may be different [10].

Research providing in-depth understanding of the behaviours of circumcised men in the general population is important in the efforts to prevent risk compensation. Kong et al. [9] recommended that more studies should focus on the population of men circumcised in programmatic settings. They also suggested focusing on behaviour changes within the short term (about six months) after circumcision [9], where such behaviour adjustments are most likely to occur. There are few qualitative studies that have explored reasons for circumcised men’s behaviour choices following circumcision [11–13]. The studies conducted in South Africa [13], Swaziland [12] and Kenya [11] provided mixed findings. The informants in these studies reported both protective and risky behaviours (the studied periods ranged from six weeks and up to 12 months). The study in Western Cape, South Africa showed that some men had intercourse before complete healing as a result of intoxication with alcohol. Others had intended to have non-penetrative sex for coping with the restriction placed on them by the wound but this escalated into intercourse. They expressed a high sexual drive during this period especially because in these densely populated townships, couples lived in cramped houses with limited interpersonal space that made it hard to avoid sexual arousal [13]. In Kisumu, Kenya and in Urban Swaziland, some men reported an increased number of sexual partners shortly after SMC and/or non-use of condoms. They felt circumcision made them sexually more desirable to women or wanted to be adventurous and have sexual experimentation in the short period after SMC [11,12]. Some also described SMC as a “back up” for condoms [12]. The men who reported HIV protective behaviours believed that behaving in a risky way would only negate the partial protection offered by SMC while some reported that it was easier to wear condoms with a circumcised penis [11,12].

There is a dearth of published studies about beliefs among SMC clients in programme settings in Uganda. One recent study in fishing communities on Lake Victoria provides some insights from clients [14]. Cultural beliefs regarding circumcision may vary in different settings and could change over time as information from SMC programmes continues to be widely disseminated. The objective of this study was to explore beliefs that may influence post circumcision sexual behaviours among circumcised adult men in a programme setting in Uganda 5 years after the SMC programme launch in the country.

## Methods

### Setting

This study was conducted in Wakiso district, central Uganda, which is contiguous to the country’s capital city. It is the most populated district in Uganda [15]. The district has 103 health facilities including four hospitals, five Health Centres (HC) IV, 37 HC III and 57 HC II offering varied services [16]. SMC services are provided free of charge at public health facilities with operational theatres such as HC IV level, and through mobile outreach clinics in areas without surgical theatres. The study was conducted among clients from five public level IV and level III HCs or their outreach points that were providing SMC services at the time of data collection for the baseline. These were in both peri-urban and rural Wakiso.

### Design and selection of participants

This qualitative description included purposively selected adult men coming for SMC at the five public HCs or outreach points run by these facilities in the district between May and June 2015. The follow-up interviews were conducted between December 2015 and January 2016. A courtesy phone call was also made to all participants in September 2015. The phone contacts of the first author were provided to all the participants, and they were free to call during the six-month period, if they had any further questions about the study. The study inclusion criteria at baseline were: age 18–59 years, ability to give written consent, married or having a stable sexual partner, seeking SMC voluntarily, and willing to be interviewed after a period of six months. Although we did not intend to stratify selection of participants by age, younger (below 25 years of age) and older participants were evenly represented. The drivers of the circumcision decisions of these men are discussed in a related paper [17]. The upper age limit (59 years) is in line with AIDS indicator survey age group while the lower age limit (18 years) is the adult age of consent in Uganda. Married men were purposely selected because they are more likely to be sexually active since exposure to sex in marriage is assumed to be higher. Study participants were recruited at the health facilities or the outreach points through health workers who informed them about the study when they came for SMC services. The research team approached the willing men and explained the details about the study before obtaining written informed consent. Participants provided their contact phone numbers and residential or other preferred addresses for the follow-up interviews. Four extra men were enrolled after the saturation point (when we concluded that no new information about beliefs was emerging [18]) to cater for potential loss to follow up. We used in-depth interviews, the best method for sensitive topics [19]. These were employed in a longitudinal strategy that also offers advantages such as building trust between the informants and the researcher over time to discuss sensitive issues in detail compared to the single snapshot interviews [20].

### Data collection and analysis

Four people (two trained male research assistants and two authors) were involved in conducting 48 in-depth interviews. The first interview in May and June 2015 with each of the informants (25 men) was held either soon after receiving the SMC service at the health facility premises, or a day after at the participant’s home, depending on what was desired. The follow-up interviews were held with 23 men at least six months after the time of circumcision, at their homes, workplaces or other private venues as they preferred. These were conducted in December 2015 and January 2016. Two men were lost to follow up; one could not be traced through his provided contacts and address, and the other declined for reasons that he did not disclose. Interviews were conducted in Luganda (district main language), Runyankore/Rukiga (spoken in south western Uganda), Lusoga (spoken in parts of eastern Uganda), and English languages, depending on what the informant preferred.

At baseline, the interview guide included the following topics: current sexual partnerships and practices regarding condom use, and expectations after circumcision. Men were also asked about any beliefs and perceptions relating to circumcision in general that they were aware of in their cultures and community as well as the sources of such beliefs. Further questions regarding the influence of such beliefs on sexual behaviour in the communities and individually were discussed. In the follow-up interviews, topics included the healing process and what was involved, resumption of sex and who they had sex with over the period, if there were any new beliefs since they were circumcised, and how these affected them, as well as perception of HIV risk. Before conducting each follow-up interview, the interviewers read the baseline interview transcript to enable better probing in case of inconsistencies in reports.

All interviews were recorded using digital voice recorders, and simultaneously transcribed and translated to English. The proof-read transcripts were then imported into atlas.ti7 qualitative data management software (ATLAS.ti GmbH, Berlin) for analysis. We used thematic network analysis as the framework for analysis [21]. Initially, inductive coding was done involving three people; two of them independent of the study planning and data collection. Initial codes were compared and discussed before a coding framework was devised and applied to the rest of the transcripts. We allowed for any emerging codes to be included for both baseline and follow-up interviews. Codes were also discussed among the first, second, and last author. Baseline and follow-up transcripts for each participant were compared to identify differences in reported behaviours, as well as beliefs. Basic themes were identified by exploring the links between the codes and clustering them. The basic themes were then arranged into organizing themes, and global themes that reflected the research question for this paper were then deduced.

### Ethical considerations

The study was approved by the Higher Degrees, Research and Ethics Committee (HDREC) of Makerere University School of Public Health (registration 288) and the Uganda National Council for Science and Technology (SS 3764). The Wakiso district health office and the health facilities where men sought SMC services granted permission for data collection. For each of the rounds of interview, the participants were compensated for their time with 20,000 Uganda shillings (about 7 US$ at the time).

## Results

### Characteristics of study participants

All participants’ demographic characteristics were collected at baseline. All men were either married or in stable sexual relationships since this was one of the study criteria. The participants’ ages ranged from 18 to 46 years, with a median age of 26 years. The majority had primary school-level education. They were from several ethnic backgrounds that are found in the Central, Western and Eastern regions of the country. Participants’ demographic details can be seen in Table 1.

**Table 1.** Participants’ characteristics

*Characteristics*	*Number of men*
**Age group**	
18–24	10
25–34	11
35+	4
**Highest education level**	
Primary	12
Secondary	9
Tertiary	4
**Residence**	
Rural	14
Urban	11
**Health facility where circumcised**	
HCIV	12
HCIII	8
Outreach	5
**Ethnicity**	
Baganda	13
Bakiga/Banyankore/Banyarwanda	5
Basoga	5
Bateso/Baluuri	2
**Occupation**	
Building/Masonry/Brick-laying/Plumbing	12
Security/barbers/taxi driving	4
Casual labour	3
Farming	3
Business/shops	3

**Figure 1.**
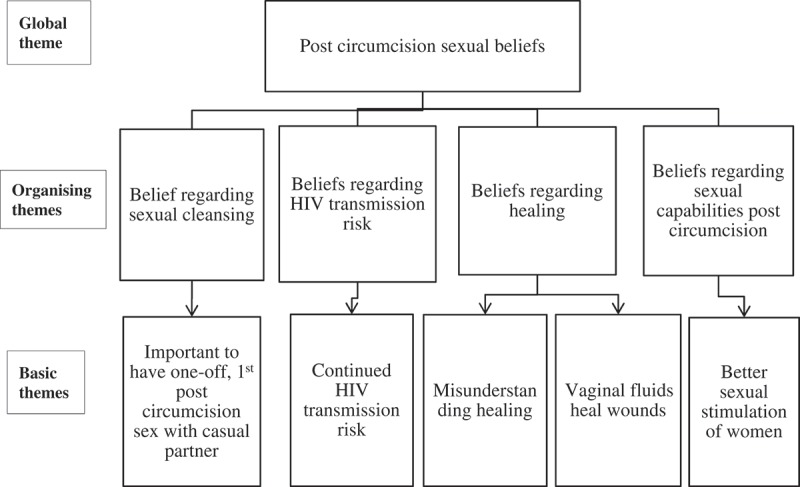
Thematic analysis for post circumcision sexual beliefs.

The overarching theme – post SMC sexual beliefs – encompassed the following organising themes [1]: Beliefs regarding sexual cleansing [2], beliefs regarding HIV transmission risk [3], beliefs regarding healing, and [4] beliefs regarding sexual capabilities post circumcision (Figure 1). The findings are presented following these themes.

### Beliefs regarding continued HIV transmission risk

Men correctly believed that although reduced, there was still a continued risk of HIV transmission after undergoing SMC. As a result of this belief, the majority of the men interviewed at follow up had either maintained or adopted safe sexual behaviour such as being faithful to one partner or use of condoms during extra marital affairs.

At follow up, twelve men reported that they still had only one sexual partner, the same partner they had at baseline. They mentioned that this was to some extent because they knew that SMC only offered partial protection, which meant they needed other measures to continue protecting themselves and their partner:

I cannot say that I have very minimal chances of getting HIV infection. I think we should not take risks just because we are circumcised. There is still a chance that you can get HIV. If you put it in your mind that you cannot get HIV, you may have trouble. If you decide to go on rampage, you may be infected in the process. Since you do not know when you may be infected, you need to be protective of yourself all the time. So you should be faithful to your one trusted partner or you should use condoms (P22, follow up, age 41).

Two men had reported extramarital affairs at baseline but both stopped casual sexual relations after SMC. Both kept their marital partners, although one had two official wives. These two attributed the behaviour change to the health education they received at the health facilities when seeking SMC services, the fear of HIV, as well as other non-SMC-related reasons as indicated in the excerpt below:

Since the time we met [referring to baseline interview] I have not had sex with any casual partner; only my wives…

I told them I have now concentrated on them and it will be them to bring any infections, not me. I was more worried about penile cancer and that is why I went for circumcision. The risk of HIV also reduced for sure… He [health worker] said circumcised men have fewer chances. But he cautioned us not to have multiple partners just because we were circumcised. Now combining my behaviour and circumcision, I should say I am better off. But I know that there is still a chance in case I have sex with anyone I am not sure of. I still have to protect myself (P10, follow up, age 46).

Three participants had multiple sexual partners following circumcision, but all reported condom use with the extra marital partner due to the belief that there was continued HIV risk. They also said that they engaged in extramarital sex because they were away from the partners for a period of time, rather than due to the circumcision state:

P: Yes, I have had sex with another woman when I went on one of my business/working trips. You know it is hard not to have these casual partners when away from home for a long period.

I: Did you use a condom with her?

P: Yes, I did. You cannot be sure about their [casual partners’] HIV status and therefore you must take caution. I am not God and I cannot determine my chances [of HIV infection]. Even though I am circumcised, I still have to use a condom. I followed what the health workers said; that in case of casual sex, we should continue to use condoms (P18, follow up, age 26).

### Beliefs regarding sexual cleansing

At both baseline and follow up, participants reported that many people believed that a circumcised man should have one-off post circumcision sex with a casual partner after healing before they have sex with their wife/stable partner. This belief was reported to be common by nearly all men in the baseline interviews and everyone in the follow up. Participants reported that they heard this from their circumcised friends, relatives, other men and women in the community, and surprisingly their sexual partners. Most reported the belief to have originated from parts of eastern Uganda where circumcision is traditionally practiced:

The first time I heard about this was from a woman actually. But she was not my sexual partner. We went to a health facility with our friend who was circumcised [in western Uganda]. This woman asked him, “now that they have circumcised you, haa! who is this woman that is going to have these ‘blades’ [perceived sharpening from the surgical blade]? If you have sex with your partner first, she is going to become a problem for you because of the ‘blades’” Then when I came to Wakiso, people were saying the same here. But I do not think it is true, surely (P1, baseline, age 25).

The reasons given for this belief varied. Most men said that they were told that the woman with whom they had initial sex would have enhanced libido and ultimately become promiscuous. The enhanced libido that a woman would experience was suspected to result from the effects of surgical blades “sharpening” the penis. Others reported that the first sexual intercourse after circumcision was for cleansing to remove a bad omen from the circumcision act, but they could not explain it further. Some also said that a woman involved in first post-circumcision sexual intercourse would become wasted and physically unattractive. Both younger and older men reported these reasons:

I have heard that you should not have first intercourse with your girlfriend after circumcision. That whoever you have first sex with will develop “akasagazi” [unusual libido]. That also her skin becomes pale and unattractive to men (P25, baseline, age 18).

In the baseline interviews, eight men (seven of them above 24 years) did not believe in this myth and the advice given by friends to comply with it. However, ten men were worried about the consequences in the event that they did not comply with this belief, and were confused about what to do when they healed. Four young men (18–24 years) were strongly inclined to find casual partners to have sex with soon after healing. The narratives below from baseline and follow up interviews are a typical example of a worried participant and his reaction:

Yesterday my friend told me that ‘now that you are circumcised, you cannot have first sex with your wife after healing.’ I told him, ‘you know that I do not have sexual partners outside my marriage. How come you are telling me to do this?’ Then he told me that there are bad omens associated with this sex, but he could not explain. I asked my other friend today if he knows this and he told me the same thing… It is good that you have come, you can help me. I am really worried. I have been wondering what will happen to my wife… My friend who told me is circumcised too. It means he had another woman outside [marriage] that he first had sex with (P21, baseline, age 29).

Then at follow up he said:

Even when I was healed, some of my friends at the construction site used to tell me that I should get someone else before having sex with my wife. But I told them that I had already had sex with her. They said that she will become promiscuous. That I should just wait for this. But up to now, I have not seen this happening. If she was promiscuous, I would have known by now. Naturally though, as any normal person you will have questions lingering in your mind, thinking about this. But I made up my mind and decided I will stick with my wife (P21, follow up, age 29).

Four young men were inclined to follow the advice they were given by those who had this belief:

I: How are you going to deal with this belief?

P: I will not have first sex with my partner. I already have another woman that I am planning to have first sex with.

I: Will she not refuse this too?

P: She will not know that I am recently circumcised (P25, baseline, age 18).

In the follow-up interviews, four men (P2, P13, P14 and P24) reported that they had first sexual intercourse post SMC with a casual partner in fear of the consequences of not complying with this belief. A fifth participant (P25) was also waiting to have such sex. Three of them were those who had indicated strong inclinations towards adhering to this belief in the baseline interviews. All the five men had primary-level education, and were aged below 25 years. Two of these young men said they had been influenced by their wives/partners to have such sex because they also worried about what would happen to them if they did not adhere to this belief. A third man had initiated sex with his wife but was consistently using condoms, waiting to find a woman that he would have condom-less sex with for cleansing. His partner, he said, had indicated that she would not accept condom-less sex until he found another woman with whom to have such intercourse. Only one of these four young men that confirmed to have sex with someone else reported using a condom during the sexual encounter with the casual partner following this belief:

P: They told us that after circumcision, it is not a good idea to start having sex with your wife or a woman you love. Haji told me this

I: Why do you think Haji said this?

P: Because he is my friend and he cares about me. I was also seeking advice from him since he had gone through this process [circumcision]. He knows my wife. He told me to look for another woman, maybe far from here… I got a partner from the past and when I healed, I went back to her again, disguising it as a resumption of the casual sexual relationship we had before. I will not have sex with her again. Hajji told me that if I do, then the bad omen will come back to me again […]

I: Sticking with this issue, did you use a condom with her on that occasion?

P: No, I did not.

I: Why did you not?

P: Haji told me that we cannot use condoms for cleansing sex (P13, follow up, age 21).

### Sexual beliefs regarding healing

This theme was organized under two beliefs: vaginal fluids aiding wound healing, and misunderstanding of healing. Four men (both younger and older) reported that vaginal fluids helped to hasten wound healing. This belief was related to wounds of different kinds, especially on the fingers. The understanding was that when vaginal fluids were applied to the wound – or if it was a finger injured, it could be inserted in a partner’s vagina- that would hasten healing. One man reported that he was told after SMC to have sexual intercourse before the wound healed to aid the healing process. However, none of the four participants believed in this, especially citing its dangers for such a sensitive body part:

P: There was a man who told me that for one to heal fast, you should have sexual intercourse. Imagine someone giving you such advice!

I: What did you do about this?

P: I could not follow this of course! How can you put your wound through such trouble? (P21, follow up, age 29).

Although most men waited to have sex until after the healing period with some taking even extra caution to wait several months, two men did not. These seemed to have misunderstood when full healing occurred, i.e. that it was more than the superficial closing of the skin, and that six weeks are needed for the inflammation to decrease and the tissue around the cut to become strong. They reported having had sexual intercourse before the recommended abstinence period ended. One had sex as soon as two weeks after surgery. Both were above 24 years of age:

P: I was not expecting this [self-ascertained quick healing]. They [health workers] had told me I will be fully healed after one month and two weeks and be able to resume sex. So I thought that is the time it will take me to heal. This [quick healing] put a smile on my face…

I: Do you mean full healing?

P: To be honest, in one week and about three days I was healed. But I waited for the full two weeks to elapse to resume sex. I was able to have sex after two weeks and I had no pain (P5, follow up, age 29)

In the discussion, both men indirectly blamed their partners as one of the reasons for engaging in early sex. One mentioned that: “There should be some tablets that women can take to reduce their sexual desire while their circumcised partners are still healing,” in reference to his partner’s sexual requests during this period. The second man who had sex before the prescribed healing period reported regrets as well as challenges of waiting, as expressed in this narrative:

I resumed sex after four weeks. Although they had told me six weeks but I was healed after four weeks ((laughs)). I thought I was okay. However, from my experience I have realised that even if they say six weeks and truly you feel healed, the first three months your skin is still weak when you have sex. I think it should be about three months or even longer if possible. The problem is that it is so hard when you are living with your partner to wait yet you see with your eyes that you are healed. You also naturally want to test how it feels after circumcision; the urge is there. Your partner is also demanding [sex] and she sees that you have healed… [But] the skin is still weak for the first three months. I used to get some sores… I got these bruises several times, but they would heal quickly (P7, follow up, age 26).

### Beliefs regarding sexual capabilities post circumcision

Participants believed that circumcision enhanced their sexual capabilities with better sexual stimulation and satisfaction for their partners. Half of the men (both young and older) had reported this belief at baseline as a part of the drivers for their circumcision decision. These also expressed a felt change at follow up after resumption of sex post SMC. Seven men further said that their partners attested to having better sexual experiences with them after undergoing SMC:

I can confirm from my sex life that now I last longer during sexual intercourse than I did before circumcision. Even my partner thanked me the first time I had sex with her [after circumcision] and was excited that I was circumcised. She had never thanked me before (P14, follow up, age 19).

One participant sustained the extramarital sexual relationship that he had before SMC. This seemed to be an experimentation with his expectations of enhanced sexual stimulation of women, which he reported in the baseline interview. “…women have a perception that a man who is circumcised is better in bed. They say so. But I am not a woman to testify to this.” Even though he reported that he had received health education at the health facility and was counselled about post SMC sexual behaviour at baseline, this did not change his behaviour. However, he noted during the follow-up interview that he was discontinuing the casual relationship:

I had a casual sexual relationship with this woman before I was circumcised. After circumcision, I also had sex with her, and she told me that I had truly changed. That she was sexually more satisfied compared to the past before circumcision. This made me happy because another woman was telling me exactly the same thing that my wife was also saying; which means it is true… But I have now decided to stick to only one partner. I will not go back to the second partner now anymore.

He continued to say:

When you are circumcised you feel better during sex and, a ‘sharpened pencil writes better than one which is not sharpened’ (P5, follow up, age 29).

## Discussion

This qualitative study explores the sexual beliefs and behaviour among men followed up six months following SMC. The study offers possible underlying explanations for the protective and sexual risk behaviours among men circumcised in a programme setting. The most commonly reported beliefs in the study were, that it was important that the initial sexual intercourse post circumcision was with someone who was not a man’s stable partner, and that circumcision offered better sexual stimulation of women. There was also some misunderstanding of what comprised complete healing, while some men had heard that vaginal fluids aided wound healing. Men also correctly believed that the risk of acquiring HIV remained even after SMC.

The findings show that some beliefs around circumcision could contribute to sexual risk behaviour. The belief that initial sexual intercourse post circumcision was intended for cleansing purposes was reported by all participants in this study. Although many participants who had heard about this misconception rejected it, some young men adhered to it, having one-off sex with casual partners, without using condoms. This should be a consideration for programme implementers because beliefs of this kind could put some newly circumcised men that adhere to them, as well as their sexual partners at the risk of HIV infection. This belief has also been reported among fishing communities on lake Victoria, Uganda [14,22] and in unpublished work in eastern Uganda [23]. It is also loosely mentioned in a national supervision report for HIV/AIDs activities [24], which may indicate that it is not only limited to this study. However, it is not mentioned in SMC social marketing documents and messages disseminated to the general public. It was also not reported to have featured during the pre-SMC counselling by the men in this study. Outside Uganda, initial post circumcision sex with casual partners has also been reported in a study in South Africa as a cleaning ritual [25].

Some beliefs around wound healing could also increase risk to HIV and other STIs. The men that reported sexual intercourse before the six weeks recommended abstinence period elapsed seemed to have misunderstood what comprised complete wound healing. Such sex has been associated with higher odds of HIV infection among circumcised men [26] and other consequences, like increased risk of infection of the surgical incision [27]. Indeed, one of these participants reported longer complete wound healing, possibly as a result. Non-adherence to the recommended healing period has also been reported elsewhere [13,28–30]. In a study by Herman-Roloff et al. in Kenya, men who reported that their sexual partners were pleased with their circumcision decision were more likely to engage in sex within the healing period [30]. The men in our study who had sex before healing had also reported at baseline that their partners were please with their decision to circumcise. The fear of partner infidelity during the healing period could also have contributed to early sex resumption. Such fears have also been reported by community members in a study in Tanzania [31]. Another reported belief related to healing was that vaginal fluids accelerate wound healing, which has also been reported in other areas of Uganda recently [14,22,23]. No man in this study reported engaging in early sex resumption for this purpose, but such misconceptions should not be ignored in SMC promotion messages to the general public.

There were also beliefs described by participants which may contribute to protective behaviour. Men believed that there was a continued risk of HIV transmission after SMC. As a result, most men either maintained or adopted safe sexual behaviour in the follow up period; having sex with only their wives or using condoms when they had extra marital sexual relations. The adherence to this correct belief could also indicate that the implementation of the SMC programme has not necessarily led to behavioural risk compensation among adult men in the general population, although this qualitative study cannot be generalized to that effect. Similar behaviours have been reported among SMC clients in Kenya [11,32] and Swaziland [12]. Some men attributed such decisions to the awareness from pre-SMC health education received at health facilities as well as public campaigns promoting SMC. These messages that emphasize partial risk reduction as opposed to complete protection seem to have positive effect on post SMC behaviour.

The belief that circumcision offered better sexual stimulation for women was also common but did not seem to result in sexual risk-taking behaviour among men in the study. Such a belief could result in sexual experimentation with casual partners after circumcision especially in the short period after healing [12], without using condoms. However, such potential risky behaviour seemed to have been neutralized among participants in this study, by the belief of continued existence of HIV risk. Nearly all participants who reported multiple sexual partners used condoms even though they expected better sexual stimulation of women after SMC.

There were other informal sources reported in this study where beliefs that may contribute to sexual risk behaviour among men arose. The belief of cleansing sex appears to be related to cultural/traditional circumcision practiced in the eastern part of Uganda as some men reported. Sexual partners of the participants, friends and other people within the community also played a role in diffusion of beliefs, while men who have gone through the circumcision experience were also consulted for advice especially in the healing period. There is limited control over such sources of information and what advice circumcised men obtain from them. Although risk compensation was not evident in this study, the existence of these beliefs that might influence some men’s post circumcision sexual behaviour should be considered in SMC programme implementation. These may be widespread. It is possible that some health workers were not aware of such beliefs at the time and missed them in the messages to SMC clients in this study. As such, these misconceptions may continue to infiltrate the communities with little control by public health authorities. Messages concerning beliefs and expected behaviours post SMC should not only target potential clients and their sexual partners, but also other influencers in the general population. This will further contribute to the success of the SMC intervention that has already reached millions of men.

There was very little variation in relation to age in reporting protective and risky beliefs, although men that adhered to beliefs leading to sexual risk behaviour were mainly of lower education levels and relatively young ages. Education generally improves knowledge and cognitive ability and is often associated with better behaviour outcomes. Men with lower education levels or no formal education may be easily influenced by such beliefs because of limited exposure to and comprehension of health promotion messages. Younger men may also be more easily influenced by their peers or by misconceptions that exist in the communities than their older counterparts. Since it may be easier for health care providers to tailor messages to young people rather than focus on client education levels, young men may benefit from further attention during health education sessions at the health facilities to allay the fears of consequences of not abiding by such beliefs. It should also be noted that most clients of the SMC intervention are likely to be young men.

The findings of this study should be regarded within the context of some limitations. The data are from self-reports of men interviewed at two time points. The follow-up data may be subject to bias for some men who may not have admitted, yet could have engaged in sexual risk behaviours. The risk of social desirability bias could have increased since at the end of the baseline interviews, men who were worried or inclined to sexual risk behaviours after healing, especially those who believed that they should have first sex with another woman, were advised against this by the interviewers and/or the first author. However, ethically we felt obliged to correct the misconceptions that would pose a risk to men after SMC and for which they had not received information from the health facilities. In-depth interviewing may have minimized this potential bias since many still reported that they adhered to the risky belief in the follow-up interviews. One of the strengths of this study is in interviewing the same clients at baseline and at six months with contact in between. Although a few studies [11–13] provide some understanding of post SMC behaviour, they were cross-sectional and with a substantial recall period of up to 12 months. Prospective qualitative studies help to build more trust with informants to discuss sensitive personal issues [20] and with shorter follow-up periods, they may yield more reliable findings and may be less prone to recall problems.

## Conclusions

This study has explored the beliefs that may influence sexual behaviour of men before and after SMC. Although most men reported to have maintained or adopted safer sexual behaviour after circumcision with the knowledge that there was continued HIV transmission risk, there were cases of sexual risk behaviour as well. Such behaviours resulted from existing beliefs or from a misunderstanding of what comprised full healing. The cultural or society beliefs that could have contributed to sexual risk behaviour in this study were not addressed in the standard counselling/health education sessions preceding SMC. Such beliefs need to be addressed because they may be widespread beyond the context of this study population and/or area. If so, they may expose some of the SMC clients to HIV infection. Using circumcised men that have adopted or maintained safer sexual behaviour to encourage others to adhere to such desired behaviour may benefit the national SMC programme. Many newly circumcised men in this study sought advice from friends that had undergone this experience, and this may be the case for other men elsewhere.
